# Diffuse Myocardial Fibrosis at Cardiac MRI in Young Adults Born Prematurely: A Cross-sectional Cohort Study

**DOI:** 10.1148/ryct.210224

**Published:** 2022-06-09

**Authors:** Christopher J. François, Gregory P. Barton, Philip A. Corrado, Aimee T. Broman, Naomi C. Chesler, Marlowe W. Eldridge, Oliver Wieben, Kara N. Goss

**Affiliations:** From the Department of Radiology, Mayo Clinic, 200 First St SW, Rochester, MN 55905 (C.J.F.); Department of Internal Medicine, University of Texas Southwestern Medical Center, Dallas, Tex (G.P.B., K.N.G.); Departments of Medical Physics (G.P.B., P.A.C., O.W.), Radiology (O.W.), Biostatistics and Medical Informatics (A.T.B.), Pediatrics (M.W.E., K.N.G.), and Medicine (K.N.G.), School of Medicine and Public Health, University of Wisconsin–Madison, Madison, Wis; and Edwards Lifesciences Foundation Cardiovascular Innovation and Research Center, University of California, Irvine, Calif (N.C.C.).

**Keywords:** MRI, Cardiac, Heart, Left Ventricle, Cardiomyopathies

## Abstract

**Purpose:**

To measure native T1 values, a marker of diffuse fibrosis, by using cardiac MRI (CMR) in young adults born prematurely.

**Materials and Methods:**

This secondary analysis of a prospective cohort study included young adults born moderately to extremely preterm and age-matched, term-born participants. CMR was performed with a 3.0-T imager that included cine imaging for the quantification of left ventricular (LV) and right ventricular (RV) volumes and function and native saturation recovery T1 mapping for the assessment of diffuse myocardial fibrosis. Values between preterm and term were compared by using the Student *t* test. Associations between T1 values and other variables were analyzed by using linear regression and multivariate regression.

**Results:**

Of the 50 young-adult participants, 32 were born preterm (mean age, 25.8 years ± 4.2 [SD]; 23 women) and 18 were born at term (mean age, 26.2 years ± 5.4; 10 women). Native T1 values were significantly higher in participants born preterm than in participants born at term (1477 msec ± 77 vs 1423 msec ± 71, respectively; unadjusted *P* = .0019). Native T1 values appeared to be positively associated with indexed LV end-diastolic and end-systolic volumes (β = 2.1, standard error = 0.7 and β = 3.8, standard error = 1.2, respectively), the RV end-diastolic volume index (β = 1.3, standard error = 0.6), and the LV mass index (β = 2.5, standard error = 0.9). Higher T1 values may be associated with reduced cardiac systolic strain measures and diastolic strain measures. Five-minute Apgar scores were inversely associated with native T1 values.

**Conclusion:**

Young adults born moderately to extremely preterm exhibited significantly higher native T1 values than age-matched, term-born young adults.

**Keywords:** MRI, Cardiac, Heart, Left Ventricle, Cardiomyopathies

Clinical trial registration no. NCT03245723

Published under a CC BY 4.0 license

*Supplemental material is available for this article.*

## Graphical abstract

**Figure:**
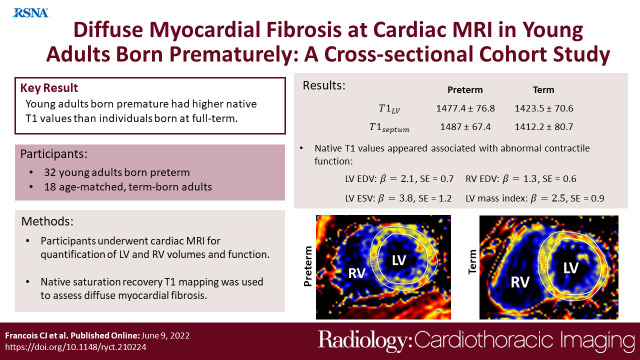

**Summary**

Young adults born moderately to extremely preterm exhibited significantly higher native T1 values than age-matched, term-born participants, suggesting that diffuse myocardial fibrosis may be present in adults born prematurely and may be associated with adverse cardiac function.

**Key Points**

■ Significantly higher native T1 values were observed in young adults born prematurely (mean native T1 value for the entire mid–left ventricular section [T1_LV_], 1477.4 msec ± 76.8; mean T1 value for a 1-cm^2^ region of interest in the septum [T1_septum_], 1487.0 msec ± 67.4; *P* = .019 and .003, respectively) compared with term-born participants (T1_LV_,1423.5 msec ± 70.6; T1_septum_, 1412.2 msec ± 80.7).
■ Increased T1 values in young adults born prematurely were associated with abnormal contractile function.

## Introduction

Preterm birth affects roughly one in 10 live births globally. With improving neonatal care practices, the majority of individuals born preterm now survive. As a result, the potential for long-term complications is increasingly recognized (1). Several studies evaluating the late cardiac effects of premature birth have found reduced biventricular chamber volumes in the preterm heart across the lifespan (2,3). A meta-analysis of studies from infancy through adulthood revealed the recovery of left ventricular (LV) contractile performance measures, including ejection fraction (EF), yet diastolic dysfunction persists (2). Among children and young adults diagnosed with heart failure, those born preterm are overrepresented, with a 17-fold increased risk being shown among those born at less than 28 weeks’ gestation (4). The risk for ischemic heart disease also appears elevated (5), although the oldest generation of extreme preterm birth (<28 weeks’ gestation) survivors is only now reaching 30–40 years of age, suggesting it may be too early to draw formal conclusions about cardiovascular disease risk in this highest-risk population.

Although prior studies have identified persistent diastolic dysfunction and increased risk of heart failure in adolescents and adults born prematurely, the presence of diffuse cardiac fibrosis and its association with cardiac function and neonatal conditions are not as well established. Cardiac MRI (CMR) can help characterize the changes in myocardial tissue in a variety of pathologic conditions. Interstitial fibrosis affects the longitudinal proton relaxation times following a preparation pulse and can be quantified by using T1 mapping sequences (6). This is distinct from the use of late gadolinium enhancement sequences to help detect areas of replacement fibrosis due to the accumulation of gadolinium-based contrast agents in areas of fibrosis (7). Native T1 values, obtained without the administration of intravenous contrast agents, are specific to the tissues being analyzed. T1 mapping is increasingly used to measure the severity and extent of diffuse interstitial fibrosis in a variety of diseases that affect the heart, including cardiomyopathies, amyloidosis, myocarditis, pulmonary hypertension, and heart failure with preserved EF (8).

The primary aim of this study was to compare native T1 values derived at CMR, a marker of diffuse interstitial fibrosis, in young adults born extremely preterm (≤32 weeks’ gestation) with those in age-matched, term-born control participants. Our primary hypothesis was that native T1 values are elevated in young adults born preterm. Secondary, exploratory aims of this study were to investigate the relationships between myocardial T1 values and measures of cardiac function and neonatal circumstances.

## Materials and Methods

### Participants

This cross-sectional cohort study was approved by the University of Wisconsin–Madison Institutional Review Board and was in compliance with the Health Insurance Portability and Accountability Act. Prior to all studies, participants gave written informed consent. Young-adult (≥18 years) participants born preterm were recruited from the Newborn Lung Project (9), a cohort of infants born at less than or equal to 32 weeks’ gestation between 1988 and 1991 in Wisconsin or Iowa with very low birth weight (<1500 g) and who were followed prospectively at the University of Wisconsin–Madison, or from the local population with the verification of a gestational age less than or equal to 32 weeks from neonatal records. A comparison cohort of age-matched, young-adult participants born at full term was recruited from the general public. Participants were free of known respiratory and cardiovascular disease. All participants did not smoke. Demographic measures collected included sex, age, current height, weight, average systolic and diastolic blood pressure, birth weight (in grams), and gestational age (in weeks).

For the participants born preterm, neonatal records were reviewed for the following: gestational age (in weeks); birth weight (in grams); 1-minute and 5-minute Apgar scores (0–10); days on mechanical ventilation, continuous positive airway pressure, and supplemental oxygen; days in the neonatal intensive care unit; the administration of maternal steroids and the presence of maternal preeclampsia (yes or no); the diagnosis of bronchopulmonary dysplasia (yes or no); the administration of neonatal steroids, surfactant, or total parenteral nutrition (yes or no); and the type of feeding during infancy (formula, breast milk, or both). This is a secondary analysis of data acquired prospectively between 2016 and 2020 (3).

The trial was registered with the United States National Library of Medicine (ClinicalTrials.gov identifier: NCT03245723).

### CMR Image Acquisition

Non–contrast-enhanced CMR was performed with a 3.0-T combined PET/MR imager (GE Signa PET/MR Discovery 750 W; GE Healthcare) (3). Native T1 mapping was assessed by using a single, short-axis, mid-LV section with a single-point saturation recovery sequence (saturation method using adaptive recovery times for T1 mapping [SMART1Map; GE Healthcare]) (10,11). Parameters for T1 mapping were 3.1-msec repetition time, 1.0-msec echo time, 350 × 350–mm^2^ field of view, 1.4 × 1.4–mm^2^ in-plane spatial resolution, and 7-mm section thickness. Right ventricular (RV) and LV size and function were assessed with multiplanar, cineangiographic, prospectively gated, balanced steady-state free precession imaging performed during end expiration. Parameters for the cineangiographic, balanced steady-state free precession were 3.1-msec repetition time, 1.1-msec echo time, array spatial sensitivity encoding technique factor = two; 2350 × 350–mm^2^ field of view, 1.4 × 1.4–mm^2^ in-plane spatial resolution, and 7-mm section thickness.

### CMR Image Analysis

Analysis of ventricular volumes, EF, mass, and native T1 values was performed by a single reader with more than 10 years of CMR experience (C.J.F.) and by using commercially available software (cvi42, version 5.11.2; Circle Cardiovascular Imaging). T1 values were calculated by using a three-parameter fit, taking the mean native T1 value for the entire mid-LV section (T1_LV_) and the mean T1 value for a 1-cm^2^ region of interest in the septum (T1_septum_) of the same mid-LV section. For the LV, end-diastolic volume (EDV), end-systolic volume (ESV), stroke volume (SV), EF, cardiac output, and mass were calculated. For the RV, EDV, ESV, SV, EF, and cardiac output were calculated. EDV, ESV, SV, cardiac output, and mass were indexed to body surface area calculated by using the Mosteller method.

Myocardial strain analysis was performed by a single reader (G.P.B.) with 5 years of CMR experience and by using commercially available software (Segment, version 2.2 R6423 strain analysis module; *http://segment.heiberg.se*) (12). Peak global longitudinal strain (GLS), global circumferential strain (GCS), and peak global radial strain (GRS) and corresponding peak global systolic strain and diastolic strain rates were calculated for the LV. Peak GLS and GCS strain and systolic strain and diastolic strain rates were calculated for the RV.

### Statistical Analysis

All measurements were tested for normality by using the Shapiro-Wilk test. Mean differences in T1 values between participants born at term and participants born preterm were compared by using the Student *t* test with equal variance. Secondary volumetric and strain measures were compared for mean differences between participants born at term and participants born preterm by using the Student *t* test; adjusted *P* values were calculated by using Hochberg methods to control for false discovery rate (FDR) (Hochberg FDR–adjusted *P* value [*P*_FDR-adjusted_]) (13). Other cardiac associations were assessed by using multiple linear regression of structural and functional cardiac measures modeled on T1 values adjusted for term or preterm status and adjusted for FDR. The effect of preterm birth versus term birth on T1 measures was tested by using a multivariate regression model adjusting for current age, sex, body mass index, and systolic blood pressure.

In participants born preterm, associations between T1 values and neonatal measures were evaluated by using linear regressions for continuous measures and adjusted for FDR. In addition, we investigated the effects of birth measures on current T1 values in a multivariate regression model, including gestational age, birth weight, the Apgar score at 5 minutes, and any ventilation (continuous positive airway pressure or mechanical) used.

The significance level was determined a priori at the .05 level, and all tests were two-tailed. Data are presented as means ± SDs or as medians and ranges, unless otherwise noted. *P* values presented for secondary volumetric and strain measures were adjusted for FDR.

## Results

### Participant Characteristics

Participant demographics are summarized in Table 1. Briefly, the preterm cohort consisted of 23 women and nine men aged 26 years ± 4, and the term cohort consisted of 10 women and eight men aged 26 years ± 5 (*P* = .75). For the preterm cohort, the gestational age was 29.2 weeks ± 2.5, and the birth weight was 1225.4 g ± 397.

**Table 1:**
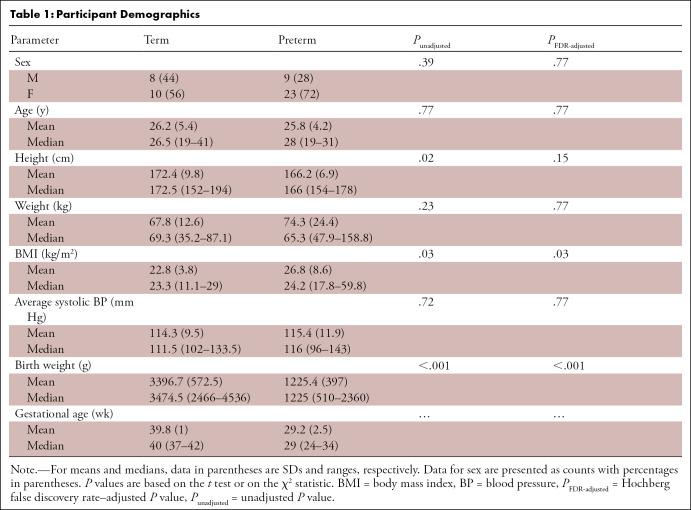
Participant Demographics

### Native T1 Mapping

**Figure 1:**
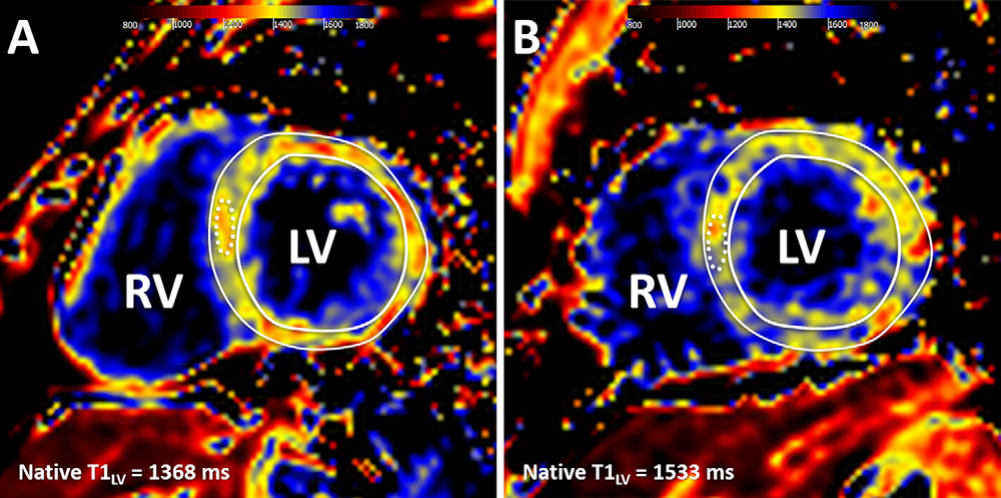
Cardiac MR native T1 mapping images in adults born **(A)** term and **(B)** preterm. Native T1 values were measured in a midventricular section with global left ventricular (LV) values recorded as a mean native T1 value for the entire mid-LV section (T1_LV_) and as a mean T1 value for a 1-cm^2^ region of interest in the septum (dotted line). RV = right ventricle.

**Figure 2:**
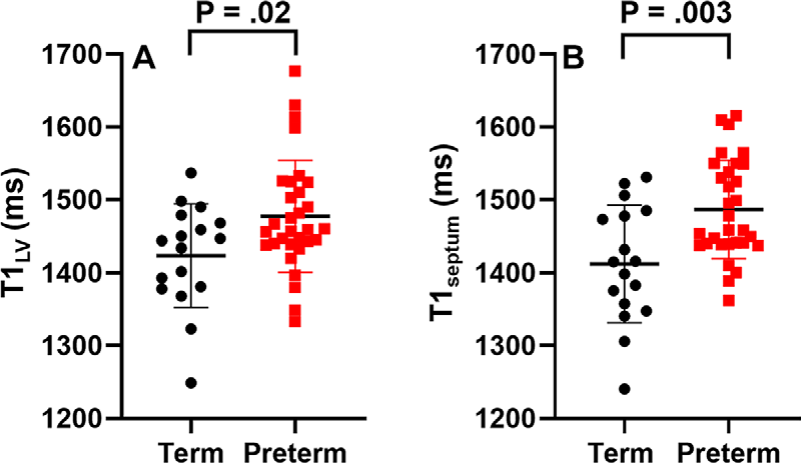
Box and whisker plots of native T1 values in young adults born at term (black circles) and young adults born preterm (red squares). **(A)** T1_LV_ and **(B)** T1_septum_ values were significantly higher in participants born preterm (mean ± SD, 1477.4 msec ± 76.8 and 1487 msec ± 67.4, respectively) than in participants born at term (mean ± SD, 1423.5 msec ± 70.6 and 1412.2 msec ± 80.7; *P* = .02 and .003, respectively). T1_LV_ = mean native T1 value for the entire mid-LV section, T1_septum_ = mean T1 value for a 1-cm^2^ region of interest in the septum.

Native T1 mapping imaging (Fig 1) was successfully performed in all participants born preterm (*n* = 32) and in all but two participants born at term (*n* = 16). In two participants born at term, native T1 mapping was not included because CMR was performed with a different MR imager (*n* = 1) or because banding artifacts precluded quantification of T1 values (*n* = 1). Native T1 values were higher in participants born preterm (Fig 2, Table 2). T1_LV_ and T1_septum_ values were 1477.4 msec ± 76.8 and 1487.0 msec ± 67.4 in participants born preterm, respectively, and were 1423.5 msec ± 70.6 (*P* = .02) and 1412.2 msec ± 80.7 (*P* = .003) in participants born at term, respectively. We found no evidence of a difference in mean native T1 values between men and women.

**Table 2:**
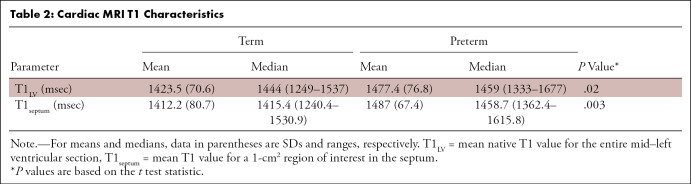
Cardiac MRI T1 Characteristics

In multivariate models adjusting for current age, sex, body mass index, and systolic blood pressure, participants born preterm had higher mean T1 values than did participants born at term; participants born preterm demonstrated T1_LV_ and T1_septum_ values of 58.3 msec (*P* = .02) and 73.4 msec (*P* = .004), respectively (Tables E1, E2 [supplement]).

### LV and RV Structure and Function

LV and RV structure and function parameters for participants born at term and participants born preterm are summarized in Table 3. Of note, indexed LV EDV, LV mass, RV EDV, and RV ESV were generally lower in young adults born preterm. For the LV, peak GLS and peak systolic GLS rate were of greater magnitude in participants born preterm than in participants born at term (−13.7% ± 2.5 and −50.7 sec^−1^ ± 11.3 compared with −11.4% ± 2.9 [unadjusted *P* value (*P*_unadjusted_) = .009, *P*_FDR-adjusted_ = .24] and −42.5 sec^−1^ ± 12.1 [*P*unadjusted = .03, *P*_FDR-adjusted_ = .63], respectively). For the RV, peak GCS and peak systolic and diastolic GCS rate were generally of greater magnitude in participants born preterm than in participants born at term (−10.6% ± 2.6, −47.8 sec^−1^ ± 14.1, and 41.2 sec^−1^ ± 13.5 compared with −8.1% ± 1.8 [*P*_unadjusted_ < .001, *P*_FDR-adjusted_ = .006], −39 sec^−1^ ± 13.2 [*P*_unadjusted_ = .04, *P*_FDR-adjusted_ = .75], and 33.2 sec^−1^ ± 12.8 [*P*_unadjusted_ = .046, *P*_FDR-adjusted_ = .75], respectively). Following FDR adjustment for multiple testing, the RV peak GCS was found to be significantly different between term and preterm groups.

**Table 3:**
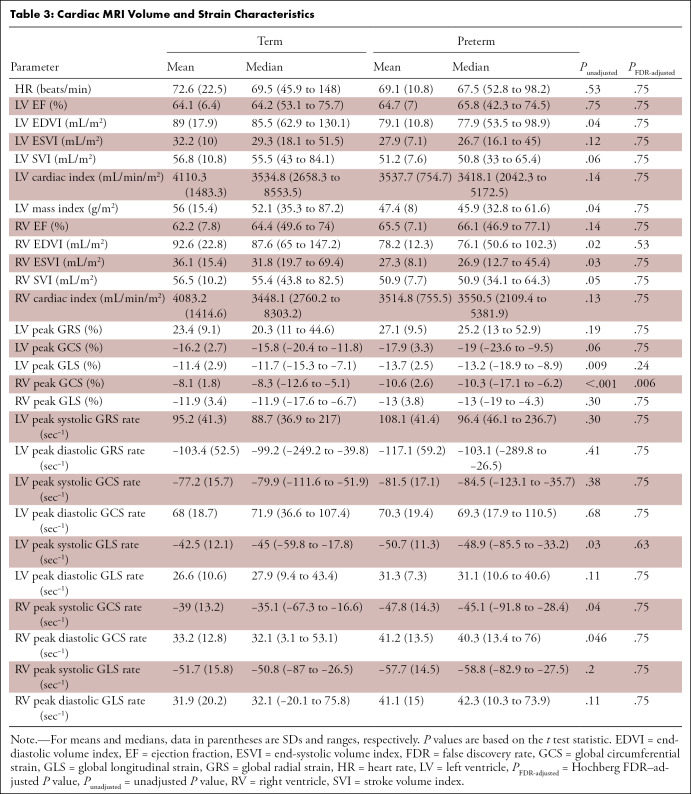
Cardiac MRI Volume and Strain Characteristics

### Cardiac Function and Native T1 Mapping Associations

Associations among CMR parameters and T1_LV_ and T1_septum_ values, adjusting for preterm status, are summarized in Table 4. After adjusting for multiple tests, no CMR parameters were significantly associated. Higher T1_LV_ values appeared to be associated with a higher LV EDV index (β = 2.1, standard error = 0.7, *P*_FDR-adjusted_ = .18), ESV index (β = 3.8, standard error = 1.2, *P*_FDR-adjusted_ = .07), mass index (β = 2.5, standard error = 0.9, *P*_FDR-adjusted_ = .25), and RV EDV index (β = 2.1, standard error = 0.7, *P*_FDR-adjusted_ = .77). Higher T1_septum_ values were observed with a higher LV EDV index (β = .06, standard error = 0.03, *P*_FDR-adjusted_ = .99), SV index (β = .04, standard error = 0.02, *P*_FDR-adjusted_ = .99), and RV SV index (β = .04, standard error = 0.02, *P*_FDR-adjusted_ = .99). In four participants born preterm with T1_LV_ values greater than 2 SDs above the mean T1_LV_ values in participants born at term, the LV EF appeared lower than in those with T1_LV_ values within 2 SDs of the mean T1_LV_ values in participants born at term (LV EF of 57.8% ± 13.1 vs 65.7% ± 5.4, respectively; *P* = .03). In three participants with T1_septum_ values greater than 2 SDs above the mean T1_septum_ values in participants born at term, we found no evidence of a difference in the LV EF relative to those with T1_septum_ values within 2 SDs of the values of participants born at term (LV EF of 65.6% ± 8.4 vs 64.6% ± 7.0, respectively; *P* = .99).

**Table 4:**
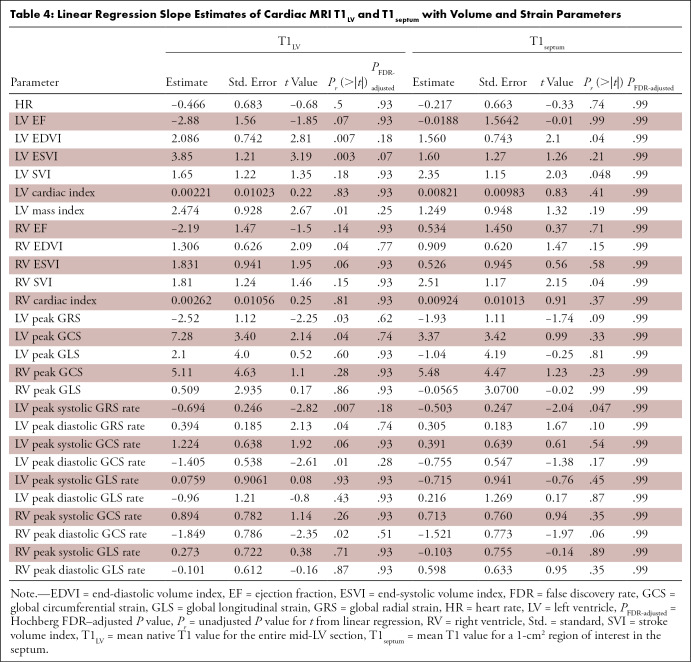
Linear Regression Slope Estimates of Cardiac MRI T1_LV_ and T1_septum_ with Volume and Strain Parameters

Higher T1_LV_ values were observed with a lower-magnitude LV peak GRS (β = −2.5, standard error = 1.1, *P*_FDR-adjusted_ = .62) and GCS (β = 7.3, standard error = 3.4, *P*_FDR-adjusted_ = .74), peak systolic and diastolic GRS rate (β = −.7, standard error = 0.2, *P*_FDR-adjusted_ = .18 and β = .4, standard error = 0.2, *P*_FDR-adjusted_ = .74, respectively), peak diastolic GCS rate (β = −1.4, standard error = 0.5, *P*_FDR-adjusted_ = .28), and RV peak diastolic GCS rate (β = −1.8, standard error = 0.8, *P*_FDR-adjusted_ = .51). Higher T1_septum_ values appeared to be associated with a lower-magnitude LV peak systolic GRS rate (β = −.2, standard error = 0.08, *P*_FDR-adjusted_ = .99).

### Neonatal Variables and Native T1 Mapping Associations

With regard to neonatal variables in participants born preterm (Table 5), the strongest association was between T1_LV_ values and Apgar scores at 5 minutes, with lower Apgar scores being associated with higher T1 values, but no neonatal values were associated with T1_LV_ values following adjustment for FDR. Weaker or no associations were present between native T1_LV_ values and T1_septum_ values and other neonatal variables. Of the 31 participants with known ventilation use at birth, only seven (23%) did not require continuous positive airway pressure or mechanical ventilation.

**Table 5:**
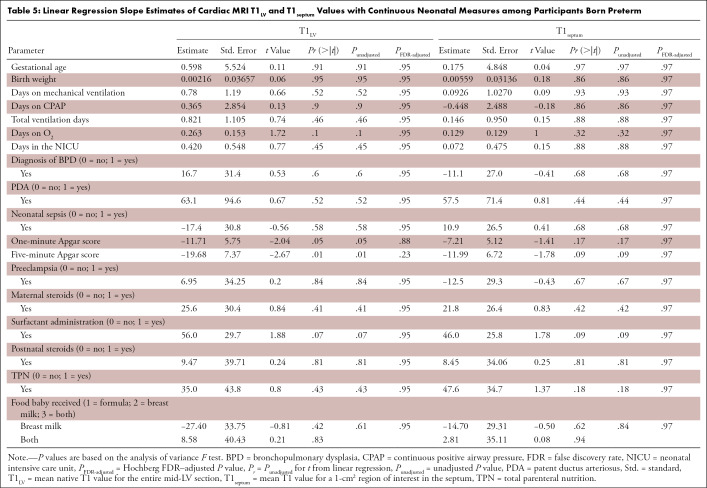
Linear Regression Slope Estimates of Cardiac MRI T1_LV_ and T1_septum_ Values with Continuous Neonatal Measures among Participants Born Preterm

In multivariate models investigating the association of birth measures and T1 values, Apgar score remained the most highly associated with T1 values, with each unit increase in an Apgar score being associated with a reduction in the T1 value (*P* = .003 for T1_LV_ and *P* = .03 for T1_septum_; Tables E3, E4 [supplement]). Gestational age, birth weight, and use of ventilation were not associated with T1 values.

## Discussion

In this study, we found that native T1 values derived at CMR were higher in adults born prematurely (T1_LV_, 1477.4 msec ± 76.8 and T1_septum_, 1487.0 msec ± 67.4) than in age-matched, term-birth participants (T1_LV_, 1423.5 msec ± 70.6; *P* = .02 and T1_septum_, 1412.2 msec ± 80.7; *P* = .003). Higher native T1 values appeared associated with larger LV and RV volumes, greater LV mass, and abnormal LV and RV strain values. Of the various neonatal variables recorded in the preterm adults, 5-minute Apgar scores showed the strongest association with T1 values.

The saturation recovery T1 mapping sequence used in this study, SMART1Map, measures the true T1 value and is less sensitive to imaging and physiologic parameters (11,14,15). As a result, SMART1Map native T1 values are longer than those obtained by using a modified Look-Locker inversion recovery sequence (10,14–16). The native T1 values in the control participants of this study, 1423 msec ± 70, are within the same range as those reported by Ferry et al (15), 1447 msec ± 45, who used the same single-point saturation recovery T1 mapping sequence at 3 T.

We found elevated T1 values, which are suggestive of diffuse myocardial interstitial fibrosis, in adults born prematurely in our study. This corroborates findings of late gadolinium enhancement on images in young adults born preterm in a prior study (17). Because our study was conducted at a single time point in young adults, it is not possible to determine whether myocardial fibrosis is present from infancy or develops over time in those born preterm. In a preclinical study of lambs born at 90% of full gestation, increased collagen deposition was present in the heart at 9 weeks’ corrected postnatal age (18), but this did not persist into adulthood (19). In another preclinical study of rodents born preterm, cardiac fibrosis developed approximately 4 weeks after hyperoxia exposure (20). Although T2-weighted imaging or T2 mapping could have helped confirm that elevated T1 values in our study were due to diffuse interstitial fibrosis rather than myocardial edema, preclinical studies have not suggested that myocardial edema is present late after preterm birth.

After adjusting for multiple comparisons, only peak GCS showed a strong difference between participants born preterm and participants born at term. Because of the relatively weak univariate relationships between T1 values and the other variables, we did not pursue any other modeling with multivariable regression. Similarly, we did not perform additional comparisons between T1 values and other CMR markers of diastolic function, such as atrial size, transmitral flow, myocardial velocities, or pulmonary venous flow. The observation of higher T1 values with a larger EDV index in our study suggests there may be remodeling with increased extracellular matrix formation in the heart of those born prematurely, which is similar to what has been reported in dilated cardiomyopathy (21,22). The apparent inverse association between elevated T1 values and abnormal strain and strain rate indexes is consistent with previous studies documenting an association between fibrosis and myocardial strain indexes in cardiac disease (23–27). The findings in our study suggest that diffuse myocardial interstitial fibrosis in young adults born prematurely may contribute to perturbed diastolic function, which is a common finding in young adults born preterm (2). Without performing endomyocardial biopsy, it is not possible to confirm this finding, and longitudinal studies in preterm individuals are needed to determine the evolution of elevated T1 values and whether baseline T1 values predict adverse outcomes as they do in patients with dilated cardiomyopathy (28).

Interestingly, we observed a possible association between 5-minute Apgar scores and T1 values, with higher T1 values occurring in participants born with lower Apgar scores. Of the five components of the Apgar score (color, heart rate, reflexes, muscle tone, and respiration), several reflect cardiac function and are reduced in infants with cardiac depression at births requiring resuscitation efforts. Low Apgar scores can predict short-term prognosis and the need for more cardiopulmonary resuscitation in the first 6–8 hours of life (29) and may predict a higher risk for respiratory morbidity after preterm birth in childhood (30). The prediction of late cardiac morbidity has not been described. Intriguingly, other metrics of more severe postnatal illness, such as the duration of ventilator or oxygen support, did not correlate with the degree of diffuse cardiac fibrosis in our study. Larger multicenter studies should be able to better address the effect of preterm birth and neonatal resuscitation on the relationship between T1 values and cardiac function in this population.

This study had several limitations. First, the relatively small size of the cohort likely contributed to the low number of significant associations. Many of the original participants in the Newborn Lung Project cohort, recruited from 1988 to 1991, have been lost to follow-up. However, we have not observed any differences in characteristics of participants born preterm from the original Newborn Lung Project cohort and those recruited de novo for this study. In addition, the absence of imaging after the administration of intravenous gadolinium-based contrast agents did not allow us to assess areas of replacement fibrosis or calculate extracellular volume. Without longitudinal data, it is not possible to determine whether the elevated native T1 values are constant or whether they change over time. Although very high temporal resolution is required to accurately measure strain rates, particularly systolic strain rates, we observed significant differences in LV peak diastolic GLS rate and RV peak systolic and diastolic GCS rates. Future studies using echography or higher temporal resolution strain imaging are necessary to confirm the presence of abnormal strain rates in those born preterm.

In conclusion, young adults born moderately to extremely preterm (≤32 weeks’ gestation) demonstrated evidence of diffuse interstitial myocardial fibrosis based on CMR native T1 mapping. The diffuse fibrosis may be associated with cardiac chamber size and function, providing additional evidence of a distinct cardiomyopathy associated with premature birth. In our analysis, lower 5-minute Apgar scores appeared to be most associated with higher native T1 values. Young adults born prematurely, particularly those with lower Apgar scores, warrant close follow-up to monitor for adverse cardiovascular outcomes.
